# Crystal Structure Prediction for Benzene Using Basin-Hopping Global Optimization

**DOI:** 10.1021/acs.jpca.1c00903

**Published:** 2021-04-21

**Authors:** Atreyee Banerjee, Dipti Jasrasaria, Samuel P. Niblett, David J. Wales

**Affiliations:** † Yusuf Hamied Department of Chemistry, University of Cambridge, Lensfield Road, Cambridge CB2 1EW, United Kingdom; ‡ Max Planck Institute for Polymer Research, 55128 Mainz, Germany; § Department of Chemistry, University of California, Berkeley, California 94609, United States; ∥ Materials Science Division, Lawrence Berkeley National Laboratory, Berkeley, California 94609, United States

## Abstract

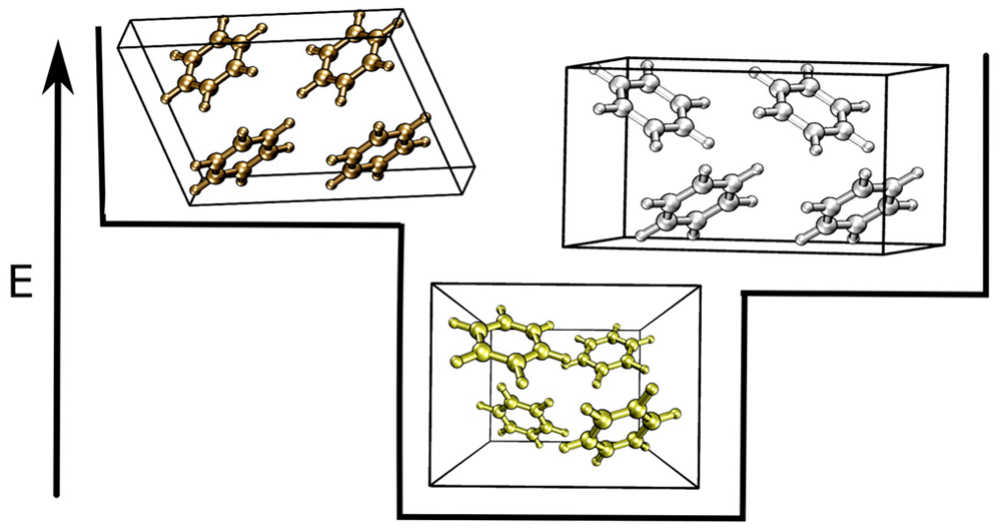

Organic molecules can be stable in distinct crystalline forms, known as polymorphs, which have significant consequences for industrial applications. Here, we predict the polymorphs of crystalline benzene computationally for an accurate anisotropic model parametrized to reproduce electronic structure calculations. We adapt the basin-hopping global optimization procedure to the case of crystalline unit cells, simultaneously optimizing the molecular coordinates and unit cell parameters to locate multiple low-energy structures from a variety of crystal space groups. We rapidly locate all the well-established experimental polymorphs of benzene, each of which corresponds to a single local energy minimum of the model. Our results show that basin-hopping can be both an efficient and effective tool for polymorphic crystal structure prediction, requiring no *a priori* experimental knowledge of cell parameters or symmetry.

## Introduction

Molecular crystals have a variety of applications in pharmaceuticals, pigments, and organic electronics. Predicting the structures of these crystals computationally can be a valuable starting point, both for understanding the physical properties of existing materials and for designing new materials.^1,2^ Enormous progress in computational crystal structure prediction (CSP) has been made over the last couple of decades. Since 1999, the Cambridge Crystallographic Data Centre (CCDC) has organized numerous CSP blind tests for organic molecules. In the early tests^3−6^ it was widely assumed that the experimental crystal structure should be the one that is most thermodynamically stable, so CSP methods focused on finding the global minimum of the potential energy landscape (PEL) for a crystal.

However, molecules can adopt different crystal structures, i.e. polymorphs, under different experimental conditions. Physical properties of polymorphs may differ significantly, with potentially dramatic consequences for the bioavailability and stability of pharmaceuticals,^7^ so a good CSP protocol should identify all the low-energy polymorphs rather than the single most-favorable structure. This requirement complicates the problem significantly because identifying multiple stable structures requires exploring high-dimensional configuration space.^1^ Most CSP methods use a physically motivated potential energy model to compute the lattice energy of candidate structures, which requires a search for low-energy regions of the PEL.

Choosing the model potential is one of the key decisions in designing a CSP protocol. Many systems can be described adequately by atom–atom isotropic potentials,^8,9^ typically with contributions from Pauli repulsion, dispersion, and electrostatic interactions. Alternatively, electronic structure calculations aim to compute the potential energy by solving for the full electronic wave function or electron density rather than by imposing an approximate model.^10^ Dispersion-corrected density functional theory (D-DFT) has proved to be particularly successful for crystal structure prediction.^11^ While these methods can be relatively accurate, they are associated with significant computational expense. Several approaches have attempted to combine the accuracy of electronic structure methods with the efficiency of classical force fields, typically by fitting a simple mathematical form to data from electronic calculations. One such model^12^ is employed in this work and is discussed in more detail below.

Several approaches have been suggested to identify crystal structures for a given model potential. Many employ molecular dynamics (MD) simulations to sample the feasible rearrangements of the crystal structure. However, the time scales associated with these solid–solid polymorphic transitions are long, and enhanced sampling techniques are needed.^13−18^ These CSP methods based on enhanced sampling risk imposing a bias on the structure search, if an order parameter is required. Recent efforts to select order parameters using artificial neural networks may help to alleviate this problem.^19,20^ Alternative approaches aim to generate candidate crystal structures without simulating dynamics, for example, by using Monte Carlo^21^ or evolutionary algorithms.^16,22,23^

Benzene is a popular system for testing and benchmarking CSP protocols. Several model potentials are known to describe benzene accurately, and the simplicity and rigidity of the benzene structure makes CSP computationally feasible, even for *ab initio* methods. Five polymorphs of benzene have been identified experimentally from X-ray and Raman spectroscopy,^24−28^ providing a challenging test for different potentials and CSP methodologies. Naming conventions for these polymorphs differ; we follow Raiteri et al.^13^ and denote them as I, II, III, III′, and IV. Under standard conditions, the orthorhombic benzene I polymorph is well understood to be the lowest-energy structure,^29^ and it has been accurately characterized using both experimental and theoretical methods.^13−15,21,30^ Monoclinic benzene III has been widely reported to be stable at higher pressures.^24,25,27,28,31,32^ Evidence for stability of the remaining three phases, structure IV at high temperatures, II at intermediate pressure, and III′ at very high pressure, is mixed.^27,28,33−35^

CSP studies of benzene using Monte Carlo simulations,^21^ metadynamics,^13^ evolutionary algorithms,^36,37^ and enhanced MD-based sampling methods^15^ have successfully located the well-known benzene I (*Pbca*) and III (*P*2_1_/*c*) structures. Most protocols find no evidence of the more controversial phases, but it remains unclear whether this absence is an artifact of incomplete or biased sampling.

In the present work, we use a rigid-body anisotropic pair potential and basin-hopping global optimization to locate crystal structures of benzene. Basin-hopping global optimization has proved effective in structure prediction for a diverse range of systems, spanning atomic and molecular clusters, glass formers, and biomolecules.^38−42^ It is an unbiased stochastic global optimization method based upon hypersurface deformation, where a local geometry optimization follows each configurational perturbation, transforming the landscape while preserving the energies of all the minima. This transformed landscape is easier to explore than the undeformed potential. We note that basin-hopping has also been used to refine quasi-random sampling in a recent contribution.^43^

We have expanded the basin-hopping global optimization method for periodic systems and used it to identify crystal structures of benzene. We employ periodic boundary conditions with dynamic cell parameters, successfully locating the lowest-energy crystal structures of benzene without any experimental information. The three most commonly reported crystal structures, benzene I, III, and III′, are rapidly located by basin-hopping global optimization for a small unit cell containing four molecules. Two additional structures, denoted benzene II′ and V, which have been identified in previous theoretical studies, have also been located using our basin-hopping approach.

The paper is organized as follows: The model potential and the basin-hopping framework for periodic, rigid body systems are described in the Methods. In the Results and Discussion we present our results and compare them with experimental data available in the literature. The Conclusions summarize our conclusions.

## Methods

### Model Potential

Much of the previous work on benzene crystal structure prediction uses isotropic model potentials,^13,15^ which assume that intermolecular forces are directionally independent. This assumption breaks down for molecules with spatially asymmetric electron densities, including benzene with its conjugated π bonds.

Instead, we model benzene using the polycyclic aromatic hydrocarbon anisotropic potential (PAHAP),^12^ in which the interaction between two atoms depends on both the distance between them and the orientations of the corresponding molecules. Molecules are treated as rigid bodies with a fixed geometry that was obtained by in vacuo optimization by density functional theory (DFT).^12^

PAHAP is a general model for polycyclic aromatic hydrocarbons, of which benzene is a special case. The atom–atom interaction potentials were parametrized by fitting to calculations using symmetry-adapted perturbation theory based on DFT, SAPT(DFT).^44−46^ The form of the site–site benzene potential and its parameters are given in the Supporting Information, with full details available in the original reference.^12^

The PAHAP model has previously been used to explore the energy landscapes of benzene clusters,^47^ but not of crystalline benzene. To facilitate this task, we implemented PAHAP within the generalized rigid body framework of the GMIN global optimization code.^48^

### Basin-Hopping Global Optimization

Basin-hopping is an efficient tool for locating low-lying minima of the PEL through exploration of a transformed landscape. At each step of the algorithm, every rigid benzene molecule was translated and rotated by a randomly selected amount, up to 0.159 Å and 0.3 radians, respectively. The energy of the perturbed structure was then minimized using the limited-memory Broyden–Fletcher–Goldfarb–Shanno (LBFGS) algorithm.^49,50^ The minimized structure was accepted or rejected according to the Metropolis criterion, comparing the new potential energy with that of the previous minimum. The fictitious temperature of the Metropolis criterion was adjusted dynamically to maintain an acceptance ratio of around 0.5. If accepted, the coordinates of the minimized structure were stored for later analysis and used as the starting point for the next step. The convergence condition for local minimization applied throughout corresponds to reduction of the root mean square gradient to 1.8897e-6 kJ/mol/Å.

The geometry optimization procedure effectively transforms the PEL into the basins of attraction^42,51^ of local minima. The minima themselves are unaffected, but downhill barriers on the landscape are eliminated, facilitating exploration of the landscape. Basin-hopping does not require *a priori* knowledge of the important conformational coordinates, nor is any knowledge of the crystal space group required. However, the algorithm does not generate a thermodynamic ensemble of structures, so additional information is required to compute thermodynamic quantities from a landscape database. Previous applications include atomic and molecular clusters,^41^ biomolecules,^52^ soft matter,^53,54^ atomic crystals,^55^ and loss function landscapes for neural networks.^56^

GMIN provides a library of global optimization tools, mostly based on basin-hopping, and a wide selection of atomic interaction potentials for which these tools may be used. Our generalized rigid body framework can convert any potential known by GMIN into a rigid-body molecular model by defining groups of atoms whose relative positions are fixed and specified in the angle-axis coordinate system.^57−59^ We implemented the PAHAP benzene model using this framework, accounting for the fact that this particular potential requires prior knowledge of atomic connectivity.

Each model system in GMIN can be simulated as a free cluster of atoms/molecules, or as a condensed phase by applying periodic boundary conditions. Most previous applications have considered large supercells to reduce finite size effects, but without enforcing symmetry constraints, these supercells inevitably become disordered and noncrystalline. To resolve this problem, we have introduced several refinements, which allow GMIN to use a simulation cell containing a small number of molecules, corresponding to only one or two primitive unit cells of the target polymorphs. This approach has two advantages: first, that the crystalline nature of the structures is automatically preserved by the periodic boundary conditions applied to a small unit cell. Second, the complexity of the PEL and computational cost of basin-hopping both increase significantly with the number of molecules, so using the smallest possible cell size reduces the cost of the CSP protocol and simplifies the subsequent analysis. In general, one would perform basin-hopping CSP for a range of cell sizes to ensure that all polymorphs have been detected. In the present case, since we know that all proposed polymorphs of benzene have either 2 or 4 molecules in their primitive unit cells, we selected a single simulation cell size, *N* = 4.

Our refinements to the GMIN procedure were as follows. First, we implemented a standard Ewald summation scheme^60,61^ to compute long-ranged electrostatic forces in reciprocal space. Parameters of this summation scheme are given in the Supporting Information. Second, our simulation cells were small enough that the repulsive and dispersive interaction radii surrounding each atom extend beyond the boundaries of the cell, so that the usual minimum-image convention would exclude certain atom pairs that should interact for an extended crystalline system. Instead, the pairwise potential *U*_*ij*_ must be summed over the periodic images of atoms *i*, *j* in neighboring unit cells. For the lattice vector **a**, interactions up to *M*_**a**_ cells distant in the **a** direction were included, where *M*_**a**_ = floor(2*r*_*c*_a*) + 1.^62^ Here a* is the magnitude of the corresponding reciprocal lattice vector, a* = (1/*V*)|**b** × **c**|, where *V* is the unit cell volume. Similarly, interactions up to *M*_**b**_ and *M*_**c**_ cells distant were included in the **b** and **c** directions, respectively. Summing over periodic images using these limits captures the entire interaction sphere of each molecule without explicitly representing the coordinates of molecules in an extended supercell. We implemented this supercell summation as an independent module within GMIN, so that it can be applied to any existing potential for which periodic boundaries are defined.

Finally, a useful CSP procedure must be able to detect multiple crystal polymorphs with different densities and space groups. To this end, we extended GMIN to optimize the unit cell parameters simultaneously with the atomic coordinates. This procedure is described in the following section. To explore crystal phase space rapidly, we performed an additional structural perturbation with every third basin-hopping step, in which the unit cell lengths and angles were randomly changed by up to 0.159 Å and 0.1 radians, respectively.

### Geometry and Unit Cell Optimization

To locate all the low-lying polymorphs in a single basin-hopping run, we must allow the cell parameters to vary during a calculation, simultaneously optimizing the unit cell lengths (*a*, *b*, and *c*) and angles (α, β, and γ) in addition to molecular positions and orientations.

To facilitate these optimizations, the atomic positions were expressed as fractions of the unit cell vectors instead of absolute coordinates. In this fractional representation, the absolute atomic positions change with variations in the unit cell size and shape. The matrix **H**, whose columns are the unit cell vectors, is used to transform between the absolute coordinates, **r**, and the fractional coordinates, ***r̅***:^63^
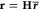
1where
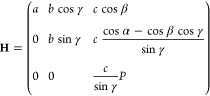
2and . The matrix **H** and its derivatives with respect to the cell parameters are well-defined, so long as the cell volume is real and positive.^64^

When considering rigid bodies in the present work, the center of mass (COM) coordinates were represented fractionally, so that the absolute positions of the molecules depend on the unit cell parameters. The angle-axis (AA) coordinates could also be represented fractionally. However, for simplicity, we assume that the molecular orientations are independent of the unit cell parameters, so the angle-axis coordinates are represented absolutely. The energy gradients with respect to absolute and fractional coordinates are not equivalent, but the gradient vanishes at a stationary point in either convention.^65^ Implementing fractional AA coordinates might alter the performance of basin-hopping slightly but would not change the underlying crystal landscape nor the qualitative results of the algorithm.

Using fractional COM coordinates and absolute AA coordinates, the position of atom *i* in rigid body *m* is
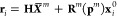
3where ***X̅***^*m*^ is the fractional position of the COM of rigid body *m*, **R**^*m*^(**p**^*m*^) is the rotation matrix derived from the AA vector, **p**^*m*^, which represents the rotation of rigid body *m* relative to a fixed reference geometry, and **x**_*i*_^0^ is the absolute position of atom *i* in that reference geometry. The reference benzene geometry used in the present work is centered at the origin and lies in the *xy*-plane of the fixed laboratory frame.

The functional form given by Equation (S2) provides the gradients of the energy with respect to the absolute rigid body coordinates directly. To optimize our rigid-body system, these gradients must be converted to the gradients with respect to fractional coordinates. For the COM coordinates, these gradients can be computed using the relation
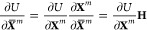
4As we chose to represent the AA coordinates absolutely, the gradients with respect to AA coordinates, ∂*U*/∂**p**^*m*^, are computed in the usual way as given in Chakrabarti et al.^47^ The derivation and expressions for the energy gradients with respect to the unit cell parameters are more complex and are given in the Supporting Information.

To prevent the unit cell from adopting physically unrealistic angle combinations during geometry optimization, the unit cell volume is constrained to be larger than zero with the use of a Weeks–Chandler–Andersen (WCA) style potential.^66^ The volume of the unit cell is
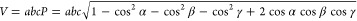
5During optimization of the cell parameters, if *P* was smaller than the dimensionless quantity σ_*P*_^1/6^ = 0.818, then a repulsive WCA term, *U*_*P*_, was added to the energy:
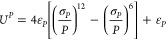
6The values for ε_*P*_ and σ_*P*_ used here were 0.001 hartree and 0.3, respectively.

## Results and Discussion

We first validated the use of the PAHAP model for crystalline benzene by analyzing experimental structures deposited at the Cambridge Crystallographic Data Centre (CCDC). These structures represent all of the well-understood benzene polymorphs. Each polymorph is represented by multiple structures with slightly different atomic coordinates, due to variations in experimental conditions.

Each experimental structure was optimized using the crystal potential energy function described above, and the resulting minima were compared to the original geometries. The energies of the minima and the CPU times required for optimization are given in Table 1. Geometric differences between the experimental and minimized structures were quantified using the root-mean-square deviation (RMSD), defined as
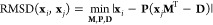
7Here **x**_*i*_ and **x**_*j*_ are two different atomic coordinate vectors. **D**, **M**, and **P** are matrices encoding the global symmetries of the system: uniform translation, rotation, and permutation, respectively. The RMSD was calculated using the Fastoverlap alignment method,^67^ an efficient algorithm for performing the minimization in eq 7. Fastoverlap aims to find the global minimum RMSD between two structures by selecting the transformation that maximizes the overlap between Gaussian functions centered on atomic coordinates of each structure.

**Table 1:** Comparison of Experimental Structures to Corresponding Local Minima of the Benzene Anisotropic Pair Potential^a^

REFCODE	energy (kJ mol^–1^)	RMSD (Å)	RMSD_15_ (Å)	LBFGS steps	CPU time/s
Benzene I (*Z* = 4)					
BENZEN	–41.587	0.131	0.074	2404	217.7
BENZEN01	–41.587	0.139	0.075	1261	182.7
BENZEN06	–41.587	0.178	0.114	582	99.2
BENZEN07	–41.587	0.145	0.082	962	117.1
BENZEN12	–41.587	0.339	0.221	1162	152.0
BENZEN13	–41.587	0.393	0.159	1249	124.4
BENZEN14	–41.587	0.178	0.115	890	122.6
BENZEN15	–41.587	0.232	0.051	1088	130.3
BENZEN18	–41.587	0.249	0.089	1277	685.9
BENZEN19	–41.587	0.155	0.083	847	126.0
BENZEN20	–41.587	0.154	0.083	943	125.2
BENZEN22	–41.587	0.375	0.292	1299	146.3
BENZEN25	–41.587	0.218	0.086	1262	683.1
BENZEN26	–41.587	0.312	0.140	1539	704.1
Benzene III (*Z* = 2)					
BENZEN16	–41.121	0.346	0.160	202	11.2
BENZEN17	–41.121	0.352	0.182	350	14.5
BENZEN21	–41.121	0.429	0.354	295	12.7
BENZEN23	–41.121	0.391	0.360	395	14.4
BENZEN24	–41.121	0.607	0.555	341	10.9
BENZEN03	–41.121	0.341	0.247	226	9.1
BENZEN04	–41.121	0.512	0.277	314	9.0
Benzene II (*Z* = 4)					
ref (71)	–40.945	1.095		613	66.4

a *Z* denotes the number of molecules in the primitive unit cell, which was always the same in the experimental and minimized structures. The second column gives the energy per molecule of each local minimum. The third column shows the RMSD per molecule between the minimized and experimental structures, and the fourth column indicates the RMSD_15_ calculated using the Mercury software.^68^ The last two columns measure optimizer performance; the CPU time to minimize the experimental structure corresponds to a single-threaded Intel Xeon X5650 CPU core running at 2.67 GHz.

As our method optimizes molecular positions and orientations and not the configuration of the rigid benzene molecule itself, we adjusted the experimental structures so that they have the same benzene geometry as the one used in our simulations. We then compared our calculated structures and the adjusted experimental structures by calculating the RMSD, which depends on the positions of all the atoms in the simulation cell, including hydrogen atoms. We have also computed the RMSD_15_ for each structure using the Mercury software.^68^ A comparison between RMSDs obtained from Fastoverlap and Mercury is given in the Supporting Information. The symmetry groups of the calculated structures were obtained using the FINDSYM software package.^69^

The RMSDs between the experimental and the minimized structures are small compared to the C–C and C–H bond lengths in most cases, confirming that minimization does not significantly alter the experimental geometry. Therefore, each CCDC structure corresponds to a nearby local minimum of the PAHAP potential. Moreover, all CCDC structures with space group *Pbca* map to a single minimum, representing the benzene I polymorph. The CCDC structures with *P*2_1_/*c* symmetry also map onto one distinct minimum, corresponding to benzene III. The range in RMSDs and optimization costs, measured in LBFGS steps, indicates that there is considerable variation between experimental structures that correspond to the same polymorph. These differences are likely due to the experimental temperature and pressure conditions as well as the experimental technique used to determine the crystal structure. We also optimized an experimental high-pressure structure reported previously^27,28^ that was not deposited in the CCDC. This structure maps to a different minimum, matching the description of benzene II. These results strongly suggest that each experimental polymorph is represented by a specific minimum on the PEL of the PAHAP model, validating this model for use in computational CSP.

**Figure 1:**
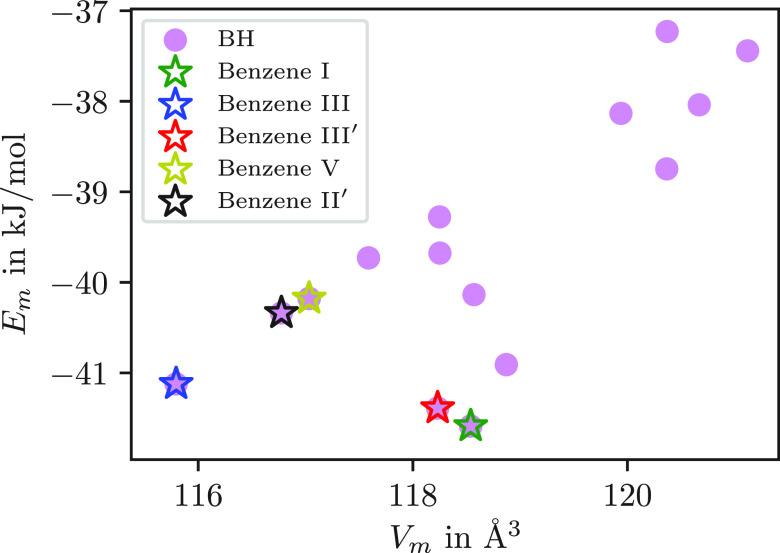
Potential energy per molecule (*E*_m_) as a function of molecular volume (*V*_m_). The stars represent the corresponding lattice energy minima of experimental benzene I,^30^ III,^32^ and III′ ^24^ structures and two structures calculated by other methods,^13,15^ all of which were also identified using basin-hopping.

We performed a series of basin-hopping calculations, each with four molecules in the simulation cell (*N* = 4) under periodic boundary conditions. Each calculation was initialized from a randomly selected high-energy structure, and no knowledge of the polymorph unit cell parameters was assumed or used to bias the calculations. The benzene potential used here supports a large number of local minima, many of which were sampled in our simulations of 10^4^ BH steps, but most of them have energies that are too high to be experimentally relevant. In Figure 1 we plot the energy per molecule as a function of molecular volume for the 15 lowest-energy minima, which have an energy per molecule that is within 5 kJ mol^–1^ of the global minimum structure. While Figure 1 shows the potential energy per molecule of the minima evaluated at *N* = 4, we checked that increasing *N* gave consistent energies and gradients to ensure that all the periodic boundary conditions were being treated correctly.

Figure 1 also shows the minima obtained by optimizing CCSD structures (labeled with the assignments given in Table 1) and two structures calculated by other CSP methods. All five structures correspond to low-lying minima that were independently identified in a single basin-hopping run, demonstrating that our algorithm is exploring configuration space effectively.

**Figure 2:**
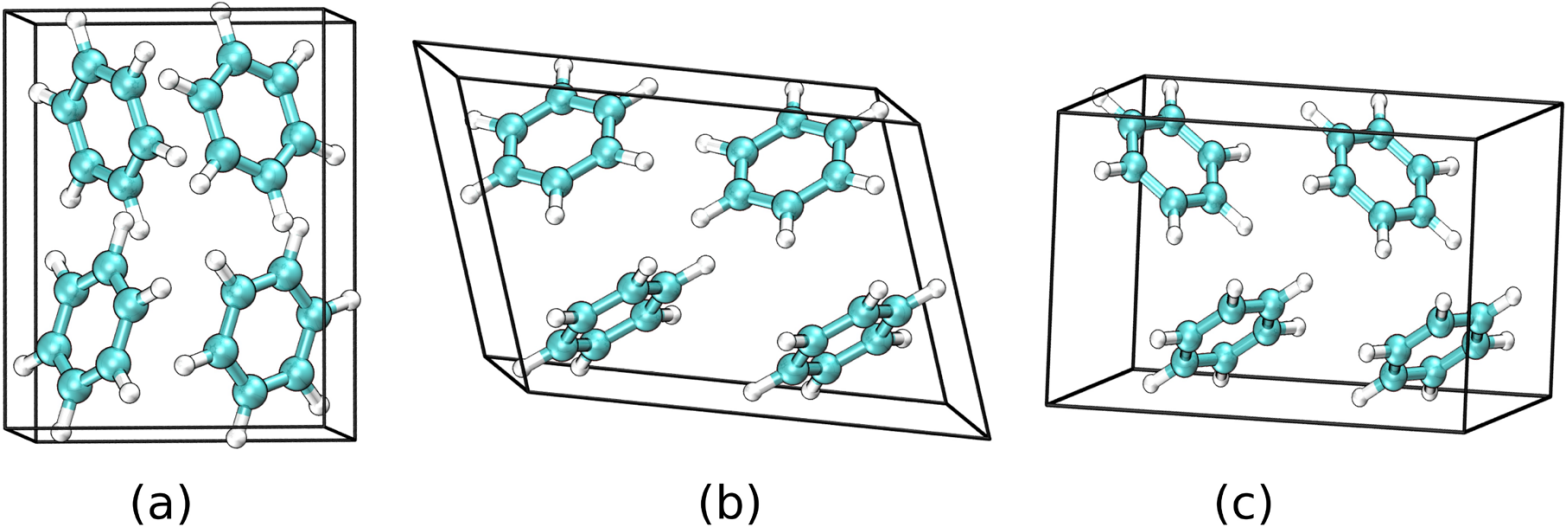
Three low-energy structures of benzene (a) I, (b) III, and (c) III′ obtained using basin-hopping with four molecules in the box (*N* = 4). Panels (b) and (c) contain two unit cells.

**Figure 3:**
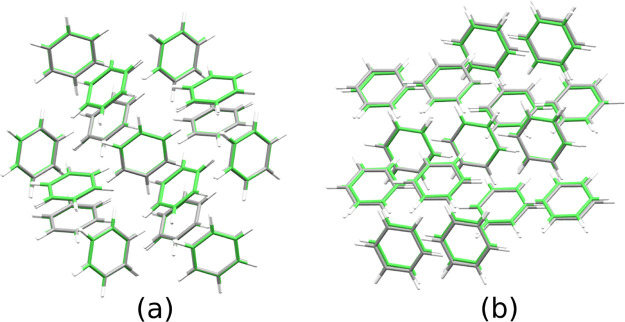
Overlays of the unit cell of the calculated (a) benzene I crystal structure (green) and refcode BENZEN (gray), with RMSD_15_ = 0.074 Å, and (b) benzene III (green) and refcode BENZEN16 (gray), with RMSD_15_ = 0.160 Å.

The minimum previously identified as the benzene I polymorph was located in every basin-hopping calculation, typically within a few steps, and always identified as the global minimum. We illustrate the primitive cell of this structure in Figure 2a. A 15-molecule supercell used to calculate the RMSD_15_ is shown in Figure 3a, overlaid with the corresponding experimental crystal structure, and the calculated cell parameters are compared with experiment in Table 2.

**Table 2:** Comparison of Crystal Structures Obtained by Basin-Hopping (BH) Global Optimization to Structures Reported in the Literature^a^

									MFET	
polymorph	*N*	*Z*	SG	*a*	*b*	*c*	β	energy	BH steps	CPU time/s
I (BH)	4	4	*Pbca*	6.868	9.477	7.285	90.0	–41.587	64	9799.6
I (experiment^30^)		4	*Pbca*	6.92	9.55	7.44	90.0			
III (BH)	4	2	*P*2_1_/*c*	5.686	11.155	7.912	112.655	–41.121	14	2003.5
III (experiment^32^)		2	*P*2_1_/*c*	5.514	5.495	7.653	110.59			
III′ (BH)	4	2	*P*2_1_/*c*	6.235	11.382	7.002	107.922	–41.389	8	1518.2
III′ (experiment^24^)		2	*P*2_1_/*c*	5.15	4.96	7.23	110.9			
V (BH)	4	4	*Pbcn*	5.367	10.241	8.741	90.0	–40.178	70	8599.7
V (theory^13^)		2	*P*2_1_	5.60	9.52	4.04	95.0			
II′ (BH)	4	4	*C*2/*c*	11.085	5.738	7.601	105.001	–40.337	90	12140.1
II′ (theory^14^)		2	*P*2_1_/*c*	5.40	5.61	7.92	106.1			

a We compare the number of molecules in the unit cell (*Z*), space group (SG), and cell parameters, with lengths given in angstroms and angles given in degrees, for each structure. All structures have α = γ = 90°. The calculated energy per molecule is given in kJ mol^–1^. The last two columns measure BH performance via the mean first encounter times (MFET) for each structure. The statistics correspond to BH runs starting from 300 high-energy structures. The standard deviation in each case is similar to the mean, and the CPU time corresponds to an Intel Xeon X5650 CPU running at 2.67 GHz.

We determined that the primitive cell contains four molecules (*Z* = 4) by calculating the distribution of angles between the molecules in the simulation cell and verifying that they were unique (see Supporting Information for more details). This value of *Z* was also confirmed using the VASPKIT software package.^70^

Our calculated structure matches well with previous reports,^13,15,21,30^ and its RMSD compared with the experimental structure is 0.13 Å per molecule, less than 10% of the benzene C–C bond length. The RMSD_15_ value between the calculated and experimental structure given by refcode BENZEN is 0.074 Å.

Benzene III was identified by basin-hopping as the third lowest minimum, and its computed structure is shown in Figure 2b. This structure has been experimentally observed at high pressure^25,28,32^ and has been successfully located in several theoretical studies.^13,15,21,31^ It has a monoclinic cell containing two molecules, which was verified in our calculated structure using the same methods as before. A periodic cell containing four benzene molecules (*N* = 4) was used for all the basin-hopping simulations in the present work, so all the figures and parameters we present correspond to a supercell containing two unit cells for benzene III. The cell parameters of the calculated and experimental structures are presented in Table 2. Optimal alignment of the experimental and calculated crystals gives an RMSD of 0.345 Å per molecule for benzene III. The RMSD_15_ between the calculated structure and the experimental structure given by refcode BENZEN16 was found to be 0.160 Å. An illustration of the overlaid structures is given in Figure 3b.

We also observed benzene III′, which has been characterized both in experiment^24^ and in simulation,^13^ as a distinct minimum. Its computed structure is shown in Figure 2c. In addition to the three experimental structures identified, basin-hopping runs located an orthorhombic minimum of benzene, which has box parameters close to those of the benzene V structure computed by Raiteri et al.^13^ (see Table 2). However, we were unable to find any experimental structure corresponding to this minimum. We have also observed a low-energy monoclinic structure (denoted as benzene II′ in Table 2) with box parameters closely resembling those reported by Schneider et al.^15^ The energy of this minimum, however, is higher than the other two monoclinic phases (benzene III and III′) that have been observed experimentally.

The polymorphs described above were all detected in every individual basin-hopping calculation, and one run would usually be sufficient to explore a crystal energy landscape. To quantify the efficiency of our algorithm, we calculate the mean first encounter time (MFET) for each polymorph. The MFET is the average time taken to locate a particular minimum in a set of independent basin-hopping runs. We used 300 runs, each starting from a distinct high-energy configuration generated in preliminary basin-hopping runs; these initial configurations had a wide distribution of unit cell lengths and angles to ensure independence of the different calculations. The value of the MFET for each structure is given in Table 2 in units of basin-hopping steps and CPU time. As expected, these quantities are directly proportional.

The MFET is less than 100 basin-hopping steps for all five polymorphs and as low as eight basin-hopping steps for benzene III, highlighting the efficiency of our approach in identifying experimentally relevant structures. The distribution of first encounter times is monotonically decreasing for all polymorphs, indicating that even short basin-hopping runs will locate most relevant polymorphs. The distributions of first encounter times for benzene I, III, and III′ are illustrated in the Supporting Information.

Surprisingly, the MFET of benzene III is smaller than that of benzene I. This result may imply that the benzene III structure, although slightly higher in energy than benzene I, occupies a larger volume of configurational space, which is located more easily by basin-hopping. This volume is related to the entropy of the polymorph, suggesting that thermodynamic effects may provide further insight.

We found that the benzene II structure^71^ (*P*4_3_2_1_2 symmetry) corresponds to a local minimum for the anisotropic pair potential. However, the energy of this minimum and its RMSD from the experimental structure are both significantly higher than for other polymorphs (see Table 1). Benzene II was not located in basin-hopping runs initialized from random configurations, but basin-hopping runs initialized from the benzene II crystal quickly located the benzene I, III, and III′ structures. This asymmetry may arise from the higher energy of the benzene II polymorph, since the global optimization runs are intended to explore low-lying minima. Our observations are consistent with some experimental studies^33,34^ that report benzene II as a metastable state. This metastability could also have an entropic contribution, which we will investigate in future work. The organization of the underlying energy landscape determines how basin-hopping explores the local minima, explaining why some minima are more easily located than others. Further insight into this landscape structure for benzene could help to optimize the basin-hopping CSP procedure for other molecular crystals.

## Conclusions

We have employed basin-hopping^38−41^ to identify low-energy structures of the benzene crystal potential energy landscape. An anisotropic pair potential for polycyclic aromatic hydrocarbons^12^ was employed using rigid bodies^58^ and periodic boundary conditions. To implement this approach, we employed Ewald summation for the computation of long-range electrostatic interactions, and we used a recently developed supercell method^62^ to calculate the supercell dimension on-the-fly. We have simultaneously optimized the rigid-body positions and orientations and unit cell parameters, allowing for crystal structure prediction with small simulation cells. Using a simulation cell containing only four molecules, our approach rapidly located the most commonly observed benzene I, III, and III′ crystal structures as well as two structures, benzene II′ and V, that have been predicted by other theoretical methods.

The use of a relatively simple molecule like benzene, which does not have the associated challenges of systems that contain both dispersion and hydrogen-bonding, allowed us to generate reliable statistics to benchmark the basin-hopping global optimization approach. By calculating the mean first encounter time for each structure, we have demonstrated the efficiency of basin-hopping in locating experimentally relevant structures for crystalline polymorphs of benzene without any biasing parameter, symmetry restrictions, or *a priori* experimental data. In future work, we will consider how the thermodynamic state influences the relative stability of polymorphs. Temperature may be incorporated in our methodology by including vibrational entropy in the free energy basin-hopping approach.^72^ Pressure can be incorporated straightforwardly in basin-hopping calculations with variable unit cells by optimizing the enthalpy *H* = *U* + *PV* in place of the potential energy *U*.^65^

We will also use our crystalline benzene framework to identify the minimum energy pathways, made up of transition states and intermediate minima, that connect different polymorph structures.^73^ These pathways will allow us to visualize the connectivity of the crystalline benzene energy landscape and to compute transition rates between the polymorphs.

## Supporting Information Available

PAHAP model and parameters, the expressions for energy gradients with respect to lattice parameters, a comparison of RMSDs computed by Fastoverlap and Mercury, primitive cell characterization, and MFET data (PDF)
