# Supplementary material for: Crystal Structure Prediction for Benzene Using Basin-Hopping Global Optimization
Source: J Phys Chem A. 2021 Apr 21;125(17):3776–84. doi: 10.1021/acs.jpca.1c00903 (PMC8279651; doi:10.1021/acs.jpca.1c00903)
Supplement: Supplementary file 1 — jp1c00903_si_001.pdf [file jp1c00903_si_001.pdf]

Supporting Information: Crystal Structure Prediction for Benzene using Basin-Hopping Global Optimization

Atreyee Banerjee,[*,†,‡,‖] Dipti Jasrasaria,[*,†,¶,‖] Samuel P Niblett,[*,†,§,¶] and David J Wales[*,†]

†*Yusuf Hamied Department of Chemistry, University of Cambridge, Lensfield Road, Cambridge CB2 1EW, United Kingdom*

‡*Max Planck Institute for Polymer Research, 55128 Mainz,Germany*

¶*Department of Chemistry, University of California, Berkeley, CA 94609*

§*Materials Science Division, Lawrence Berkeley National Laboratory, Berkeley, CA 94609*

‖*Contributed equally to this work*

E-mail: batreyee89@gmail.com; djasrasaria@berkeley.edu; sniblett@lbl.gov; dw34@cam.ac.uk

Model details

Full details of PAHAP are given in the references.[S1] The total interaction energy, U, is a sum over all pairwise interactions between molecules I and J:

$$U = \sum_{I=1}^{N-1} \sum_{J=I+1}^{N} \sum_{i\in I}^{1,12} \sum_{j\in J}^{1,12} U_{ij}(r_{ij}, \Omega_{ij})\,, \tag{S1}$$

where $U_{ij}(r_{ij}, \Omega_{ij})$ is an atom-atom pair potential. r_{ij} is the distance between sites i and j, and Ω_{ij} represents their relative orientation.

The benzene molecules are assumed to be rigid and are described using the angle-axis framework. Each benzene is a 12-site rigid body with D_{6h} symmetry, and the fixed C–C and C–H bond lengths are taken to be 1.397 Å and 1.087 Å, respectively.

The PAHAP pair potential U_{ij} is given by:

$$U_{ij}\left(r_{ij}, \Omega_{ij}\right) = G \exp\left[-\alpha_{ij}\left(r_{ij} - \rho_{ij}\left(\Omega_{ij}\right)\right)\right] - f_6\left(r_{ij}\right)\frac{C_{6,\mathrm{iso}}}{r_{ij}^6} + \frac{q_i q_j}{r_{ij}}\,. \tag{S2}$$

The first term is a Born-Mayer function that describes short-range anisotropic interactions. G is a constant set at 10^{-3} Hartree, and α_{ij} controls the hardness of the short-range interaction. $\rho_{ij}(\Omega_{ij})$ is the sum of shape functions[S2] for the individual sites i and j:

$$\rho_{ij}(\Omega_{ij}) = \rho^i(\theta_i) + \rho^j(\theta_j)\,, \tag{S3}$$

where the shape function, $\rho^i(\theta_i)$, represents the effective radius of site i:

$$\rho^i(\theta_i) = \rho^i_{00} + \rho^i_{10}\cos\theta_i + \frac{1}{2}\rho^i_{20}(3\cos^2\theta_i - 1)\,. \tag{S4}$$

Here, θ_i is the angle between the vector connecting sites i and j and the vector pointing radially outward from the carbon to the attached hydrogen. These angles are responsible for the orientational dependence of U_{ij}. The constants, ρ_{κ_i}, depend on the atom types of the

sites in the interaction.

The second term of Equation (S2) describes isotropic, damped dispersion interactions. $C_{6,\text{iso}}$ is a constant, $f_6(r_{ij})$ is a damping function, which takes the Tang-Toennies form:[S3]

$$f_n(r_{ij}) = 1 - \exp(-\beta r_{ij}) \sum_{k=0}^{n} \frac{(\beta r_{ij})^k}{k!}\,, \tag{S5}$$

where β is a constant equal to 1.6485 a_0^{-1}, and a_0 is the Bohr radius. Finally, the third term of Equation (S2) is the Coulomb potential for electrostatic interactions.

The parameters for the different types of atom-atom interactions are given in the supporting information (Table S1), and the atomic charges of carbon and hydrogen are assigned to be $-0.1114e$ and $+0.1114e$, respectively.

Non-bonded interactions in the pair potential U_{ij} were truncated at a cutoff radius of $r_c = 7.938$ Å. For distances $r_{ij} > r_c$ the anisotropic and damped dispersion terms are negligible, but to avoid even a small energy discontinuity at the cutoff we subtracted a constant value $U_{ij}(r_c)$ from each pairwise energy term. As discussed in the Methods section, this cutoff radius was often longer than the side length of the simulation box, so U_{ij} was summed over multiple periodic images of i and j to capture all interacting atom pairs.

Similarly, the electrostatic term in U_{ij} is slow to converge with r_{ij}, and the cutoff distance necessary to truncate this term without a significant discontinuity is so large that computing this term directly becomes intractable. Instead, we implemented Ewald summation,[S4,S5] which divides the long-range interaction into a short-range contribution that is calculated in real space and a long-range contribution that is calculated using a Fourier transform. This approach enables the evaluation of pairwise interaction sums to high accuracy and at a reasonable computational cost, while avoiding artifacts introduced by approximate truncation methods.

Three parameters control the behavior of the Ewald summation. The real-space cutoff and number of reciprocal lattice vectors included in the Fourier-space contribution were set

to $r_c = 7.938$ Å and $k_{\max} = 5$, respectively, ensuring that the estimated errors in the Ewald summation terms are less than 0.01 kJ/mol.[S6] The last parameter is α, which dictates the contribution of the electrostatic potential that is computed in real space. The potential is invariant with respect to α, which can be chosen to optimize performance. We chose a value of $\alpha = 5.6/L$, where L is the shortest unit cell length in the initial structure for basin-hopping, which has been suggested as an optimal value for systems with fewer than 1000 charges.[S7]

Parameters for anisotropic potential

Table S1 presents the parameters for the anisotropic potential, derived from first principles by Totton *et al.*[S1] Each pair of atoms has a hardness parameter α_{ij} and dispersion parameter C_6 that depend only on the atom types of i and j. The shape function for atom i has 2 terms if i is H or 3 terms if i is C. The coefficients ρ_{κ_i} of these terms are indexed by κ_i.

Table S1: Parameters for the pairwise potential. The first column indicates to which pair of elements each parameter set refers. For each element set, the different columns indicate different terms in the shape function expansion. All values are given in atomic units.

ij	κ_i	κ_j	ρ_κ	α_{ij}	C_6
CC	00		5.8147	1.8615	30.469
	10	10	0.0217		
	20	20	−0.2208		
CH	00	00	5.1505	1.7756	12.840
	10		0.0217		
		10	−0.2718		
	20		−0.2208		
HH	00		4.4862	1.4312	5.359
	10	10	−0.2718		

Energy gradients with respect to lattice parameters

Exploring the potential energy landscape (PEL) while optimizing the unit cell parameters also requires the gradients of the energy with respect to these six variables. Given that the total interaction energy, U, is a sum over all site-site interactions, the gradients with respect to unit cell parameters can be computed by summing the contributions from each interaction:

$$\frac{\partial U}{\partial h_\alpha} = \sum_{i>j} \frac{\partial U_{ij}}{\partial h_\alpha}\,, \tag{S6}$$

where i and j are indices of the sites in the system, and h_α is one of the six unit cell parameters.

The gradient contribution from each interaction pair can be computed using the following relation:

$$\frac{\partial U_{ij}}{\partial h_\alpha} = \frac{\partial U_{ij}}{\partial r_{ij}} \frac{\partial r_{ij}}{\partial \Delta \mathbf{r}_{ij}} \cdot \frac{\partial \Delta \mathbf{r}_{ij}}{\partial h_\alpha}\,, \tag{S7}$$

where r_{ij} is the absolute distance between sites i and j and $\Delta\mathbf{r}_{ij}$ is the absolute displacement vector between sites i and j. Note that $\partial U_{ij}/\partial h_\alpha$ and $\partial U_{ij}/\partial r_{ij}$ are scalar values, while $\partial r_{ij}/\partial \Delta\mathbf{r}_{ij}$ and $\partial \Delta\mathbf{r}_{ij}/\partial h_\alpha$ are vectors of length three.

$\partial U/\partial r_{ij}$ is calculated directly from the potential. $\partial r_{ij}/\partial \Delta\mathbf{r}_{ij} = \Delta\mathbf{r}_{ij}/r_{ij}$ due to the relationship between the absolute site-site distance and the absolute site-site displacement vector:

$$r_{ij}^2 = \Delta\mathbf{r}_{ij,x}^2 + \Delta\mathbf{r}_{ij,y}^2 + \Delta\mathbf{r}_{ij,z}^2\,. \tag{S8}$$

Finally, given the definition of absolute coordinates in Equation (3) of the main text, the absolute site-site displacement vector can be computed as:

$$\Delta\mathbf{r}_{ij} = \mathbf{H}(\bar{\mathbf{X}^m} - \bar{\mathbf{X}^n}) + \mathbf{R}^m\mathbf{x}_i^0 - \mathbf{R}^n\mathbf{x}_j^0 \,. \tag{S9}$$

The only dependence of the absolute site-site displacement vector on the cell parameters is through the matrix $\mathbf{H}$, so:

$$\frac{\partial\Delta\mathbf{r}_{ij}}{\partial h_\alpha} = \frac{\partial\mathbf{H}}{\partial h_\alpha}(\bar{\mathbf{X}^m} - \bar{\mathbf{X}^n}) \,. \tag{S10}$$

Therefore, the gradients with respect to cell parameters are:

$$\frac{\partial U}{\partial h_\alpha} = \sum_{i>j} \frac{\partial U_{ij}}{\partial r_{ij}} \frac{\Delta\mathbf{r}_{ij}}{r_{ij}} \cdot \left(\frac{\partial\mathbf{H}}{\partial h_\alpha}(\bar{\mathbf{X}^m} - \bar{\mathbf{X}^n})\right) . \tag{S11}$$

This equation is used to compute the contributions to the cell parameter derivatives from the anisotropic repulsion, damped dispersion, and real-space Ewald sum energies.

The contributions from the Fourier-space Ewald sum energy are more complicated, due to their dependence on the reciprocal lattice vectors. The lattice vectors, $\mathbf{l}$, are written as combinations of the unit cell vectors, $\mathbf{h}$, which are the columns of the matrix $\mathbf{H}$ defined in Equation (2) of the main text:

$$\mathbf{l} = n_1\mathbf{h}_1 + n_2\mathbf{h}_2 + n_3\mathbf{h}_3 \,. \tag{S12}$$

It is similarly possible to define the reciprocal lattice vectors, $\mathbf{k}$, as combinations of the reciprocal unit cell vectors:

$$\mathbf{k} = \ell_1\mathbf{b}_1 + \ell_2\mathbf{b}_2 + \ell_3\mathbf{b}_3 \,, \tag{S13}$$

where the reciprocal unit cell vectors are defined in terms of the unit cell vectors and volume:

$$\mathbf{b}_1 = 2\pi\frac{\mathbf{h}_2 \times \mathbf{h}_3}{V} \,, \text{etc.} \tag{S14}$$

The matrix $\mathbf{B}$ is defined to have columns that are the reciprocal unit cell vectors. It can be differentiated directly with respect to the unit cell parameters.

The Fourier-space part of the Ewald sum, which computes the energy due to the long-range part of electrostatic interactions, is defined as a sum over the non-zero reciprocal lattice vectors:

$$U^f = \frac{2\pi}{V} \sum_i{}^{\prime} \frac{1}{k_i^2} \exp(-k_i^2/4\alpha) \left[\Big(\sum_j q_j \cos(\mathbf{k}_i \cdot \mathbf{r}_j) \Big)^2 + \Big(\sum_j q_j \sin(\mathbf{k}_i \cdot \mathbf{r}_j) \Big)^2 \right]. \tag{S15}$$

This energy depends on the unit cell parameters through the unit cell volume, V, the reciprocal lattice vectors, $\mathbf{k}$, and the absolute position of the atoms, $\mathbf{r}$. Therefore, the gradients with respect to the cell parameters, h_α, can be computed using the following relation:

$$\frac{\partial U^f}{\partial h_\alpha} = \frac{\partial U^f}{\partial V} \frac{\partial V}{\partial h_\alpha} + \sum_i \left[\frac{\partial U^f}{\partial k_i} \frac{\partial k_i}{\partial h_\alpha} + \sum_j \frac{\partial U^f}{\partial (\mathbf{k}_i \cdot \mathbf{r}_j)} \frac{\partial (\mathbf{k}_i \cdot \mathbf{r}_j)}{\partial h_\alpha} \right]. \tag{S16}$$

The partial derivatives of the Fourier-space Ewald sum energy with respect to the unit cell volume, V, the magnitude of the reciprocal lattice vector, k_i, and the dot product of the reciprocal lattice vector with the atomic position, $(\mathbf{k}_i \cdot \mathbf{r}_j)$, are straightforward to obtain directly from Equation (S10). Determining the partial derivatives of the volume with respect to unit cell parameters is also straightforward.

The other terms are slightly more complicated due to the dependence of the reciprocal lattice vectors on the unit cell parameters, and they are defined as follows.
The squared magnitude of the reciprocal lattice vector, k^2, is:

$$k^2 = \sum_p \Big(\sum_q \ell_q \mathbf{B}_{pq} \Big)^2, \tag{S17}$$

where $\mathbf{B}_{pq}$ is the element of the reciprocal lattice matrix in the pth row and the qth column,

and ℓ_q is the integer coefficient for the qth column of the $\mathbf{B}$ matrix.

Using this relation, the partial derivatives with respect to unit cell parameters are:

$$\frac{\partial k_i}{\partial h_\alpha} = \frac{1}{k_i}\Big(\sum_q \ell_q \mathbf{B}_{pq}\Big) \cdot \Big(\sum_q \ell_q \frac{\partial \mathbf{B}_{pq}}{\partial h_\alpha}\Big)\,. \tag{S18}$$

Similarly, the dot product of the reciprocal lattice vector and absolute atomic position, $(\mathbf{k}_i \cdot \mathbf{r}_j)$ is:

$$(\mathbf{k}_i \cdot \mathbf{r}_j) = \Big(\sum_q \ell_q \mathbf{B}_{pq}\Big) \cdot \mathbf{r}_j\,, \tag{S19}$$

where $\mathbf{r}_j = \mathbf{H}\bar{\mathbf{X}}^m + \mathbf{R}^m \mathbf{x}_j^0$.

Therefore, the relevant partial derivatives are:

$$\frac{\partial(\mathbf{k}_i \cdot \mathbf{r}_j)}{\partial h_\alpha} = \Big(\sum_q \ell_q \frac{\partial \mathbf{B}_{pq}}{\partial h_\alpha}\Big) \cdot \mathbf{r}_j + \Big(\sum_q \ell_q \mathbf{B}_{pq}\Big) \cdot \Big(\frac{\partial \mathbf{H}}{\partial h_\alpha}\bar{\mathbf{X}}^m\Big)\,. \tag{S20}$$

Finally, we require a lattice-parameter gradient for the repulsive WCA potential, which was added in the Methods section of the main text to avoid sampling unphysical combinations of cell angles during basin-hopping. This derivative is given by

$$\frac{\partial U^P}{\partial h_\alpha} = 24\frac{\varepsilon_P}{\sigma_P}\Big[\Big(\frac{\sigma_P}{P}\Big)^7 - 2\Big(\frac{\sigma_P}{P}\Big)^{13}\Big]\frac{\partial P}{\partial h_\alpha}\,. \tag{S21}$$

All of these gradients with respect to lattice parameters were derived analytically, validated against the two-sided numerical gradients, and implemented in the GMIN basin-hopping code.[S8]

Comparison of RMSD from FASTOVERLAP and $RMSD_{15}$ from Mercury software

The root-mean-square deviations (RMSDs) between experimental structures and the minimized geometries were computed using the Fastoverlap alignment method,[S9] and the $RMSD_{15}$ values were computed using the Mercury software.[S10] The RMSD values for each experimental structure are shown in Fig. S1. The agreement between both methods is favorable.

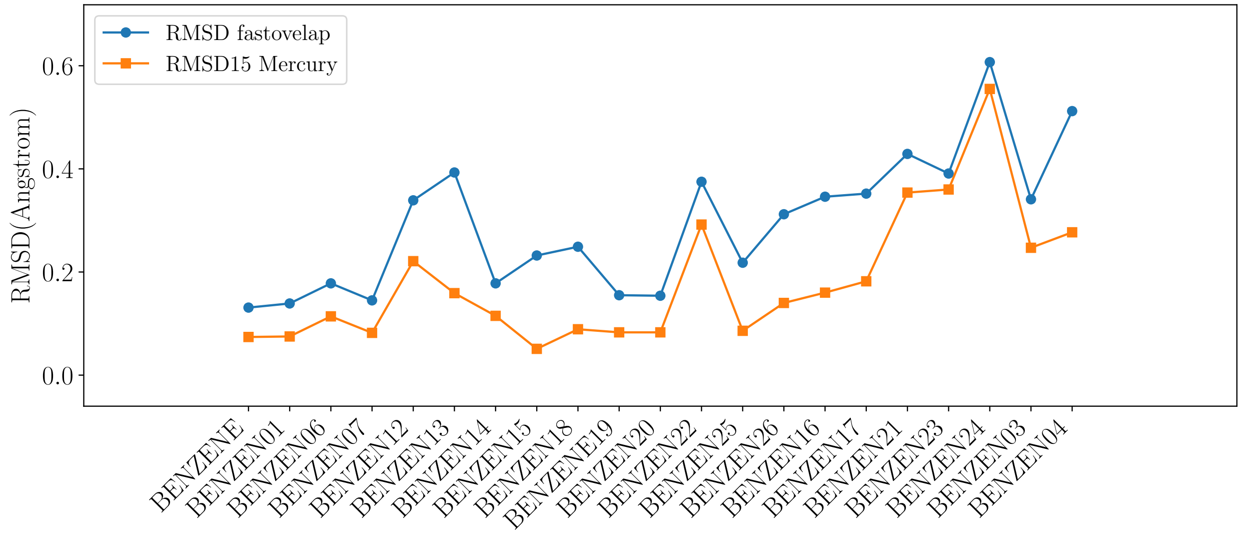

Figure S1: Comparison of RMSD per molecule using FASTOVERLAP and Mercury software ($RMSD_{15}$).

An equivalent description of number of molecules in the primitive cell

To determine an analog for the number of molecules in the primitive cell (Z) of each predicted structure, which was simulated with $N = 4$ molecules, we analyzed the distribution of relative orientations of the molecules. To characterize the orientation of the benzene molecules, we

defined the angle (θ) between the planes of two molecules in the simulation box:

$$\theta = \cos^{-1}\left(\frac{\mathbf{n}_1 \cdot \mathbf{n}_2}{|\mathbf{n}_1||\mathbf{n}_2|}\right), \tag{S22}$$

where $\mathbf{n}_1$ and $\mathbf{n}_2$ are the normal vectors to the planes containing the two molecules.

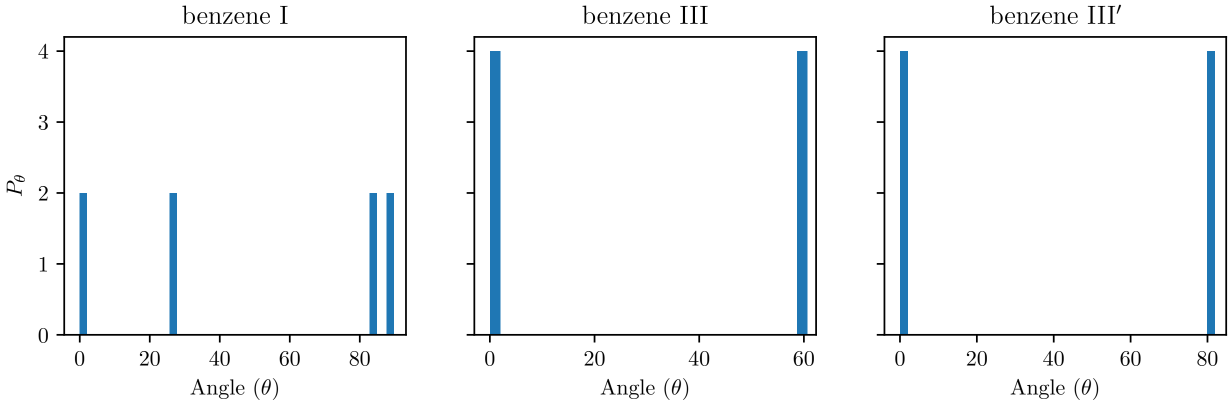

Figure S2: The distribution of angles, in degrees, between benzene molecules shows that the primitive cells contains 4, 2, and 2 molecules in benzene I, benzene III, and benzene III′, respectively.

In Figure S2 we plot the distribution of θ (P_θ) for benzene I, III, and III′, which show 4, 2, and 2 distinct values, respectively. This indicates that the primitive cells for benzene III and III′ have $Z = 2$ molecules, and the simulation boxes for those structures contain two unit cells. We have verified our result using VASPKIT software package.[S11]

Mean first encounter time (MFET)

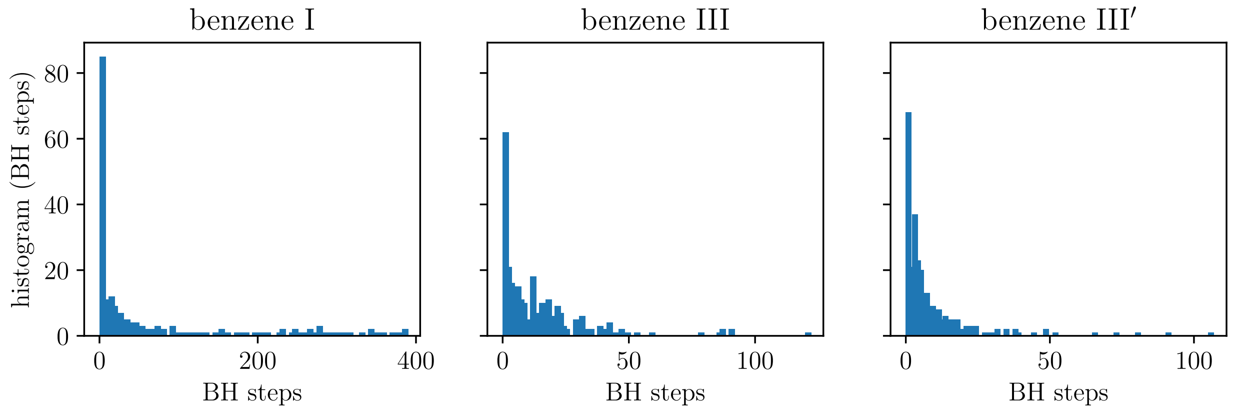

Figure S3: The histogram of BH steps required to obtain benzene I, benzene III, benzene III′ structures, respectively. The distribution shows Poissonian-type behavior of first encounter times. The histogram was calculated using around 300 calculations for each structure.

References

(S1) Totton, T. S.; Misquitta, A. J.; Kraft, M. A first principles development of a general anisotropic potential for polycyclic aromatic hydrocarbons. *J. Chem. Theory Comput.* **2010**, *6*, 683–695.

(S2) Stone, A. J. *The Theory of Intermolecular Forces*; Oxford University Press, 2000.

(S3) Tang, K. T.; Toennies, J. P. An improved simple model for the van der Waals potential based on universal damping functions for the dispersion coefficients. *J. Chem. Phys.* **1984**, *80*, 3726–3741.

(S4) Ewald, P. P. Die Berechnung optischer und elektrostatischer Gitterpotentiale. *Ann. Phys. (Berlin, Ger.)* **1921**, *369*, 253–287.

(S5) Frenkel, D.; Smit, B. *Understanding molecular simulation: from algorithms to applications*; Elsevier, 2001; Vol. 1.

(S6) Kolafa, J.; Perram, J. W. Cutoff errors in the Ewald summation formulae for point charge systems. *Mol. Sim.* **1992**, *9*, 351–368.

(S7) Rycerz, Z.; Jacobs, P. Ewald summation in the molecular dynamics simulation of large ionic systems: The cohesive energy. *Mol. Sim.* **1992**, *8*, 197–213.

(S8) Wales, D. J.; Bogdan, T. V. *GMIN: A program for finding global minima and calculating thermodynamic properties from basin-sampling*; http://www-wales.ch.cam.ac.uk/GMIN.

(S9) Griffiths, M.; Niblett, S. P.; Wales, D. J. Optimal alignment of structures for finite and periodic systems. *J. Chem. Theory Comput.* **2017**, *13*, 4914–4931.

(S10) Macrae, C. F.; Sovago, I.; Cottrell, S. J.; Galek, P. T.; McCabe, P.; Pidcock, E.; Platings, M.; Shields, G. P.; Stevens, J. S.; Towler, M. Mercury 4.0: From visualization to analysis, design and prediction. *J. Appl. Crystallogr.* **2020**, *53*, 226–235.

(S11) Wang, V.; Xu, N.; Liu, J. C.; Tang, G.; Geng, W.-T. VASPKIT: A user-friendly interface facilitating high-throughput computing and analysis using VASP code. *arXiv preprint arXiv:1908.08269* **2019**,
